# IL-18–secreting CAR T cells targeting DLL3 are highly effective in small cell lung cancer models

**DOI:** 10.1172/JCI166028

**Published:** 2023-05-01

**Authors:** Janneke E. Jaspers, Jonathan F. Khan, William D. Godfrey, Andrea V. Lopez, Metamia Ciampricotti, Charles M. Rudin, Renier J. Brentjens

**Affiliations:** 1 Department of Medicine, Memorial Sloan Kettering Cancer Center, New York, New York, USA.; 2 Weill Cornell School of Medicine, New York, New York, USA.; 3 Thoracic Oncology, Memorial Sloan Kettering Cancer Center, New York, New York, USA.; 4 Department of Medicine, Roswell Park Comprehensive Cancer Center, Buffalo, New York, USA.

**Keywords:** Immunology, Oncology, Cancer immunotherapy, Cellular immune response, Lung cancer

## Abstract

Patients with small cell lung cancer (SCLC) generally have a poor prognosis and a median overall survival of only about 13 months, indicating the urgent need for novel therapies. Delta-like protein 3 (DLL3) has been identified as a tumor-specific cell surface marker on neuroendocrine cancers, including SCLC. In this study, we developed a chimeric antigen receptor (CAR) against DLL3 that displays antitumor efficacy in xenograft and murine SCLC models. CAR T cell expression of the proinflammatory cytokine IL-18 greatly enhanced the potency of DLL3-targeting CAR T cell therapy. In a murine metastatic SCLC model, IL-18 production increased the activation of both CAR T cells and endogenous tumor-infiltrating lymphocytes. We also observed an increased infiltration, repolarization, and activation of antigen-presenting cells. Additionally, human IL-18–secreting anti-DLL3 CAR T cells showed an increased memory phenotype, less exhaustion, and induced durable responses in multiple SCLC models, an effect that could be further enhanced with anti–PD-1 blockade. All together, these results define DLL3-targeting CAR T cells that produce IL-18 as a potentially promising novel strategy against DLL3-expressing solid tumors.

## Introduction

Small cell lung cancer (SCLC) accounts for about 15% of lung cancers, and two-thirds of cases are metastatic at the time of diagnosis (1). Despite initial sensitivity to chemotherapy, metastatic SCLC almost always recurs, leading to a median overall survival of approximately 13 months (2). The addition of immune checkpoint blockade (ICB) has not led to a similar benefit as that seen in other cancers with high tumor mutation burdens, such as melanoma (3), a difference that might be attributable to the notably low MHC-I expression on SCLC cells (4, 5). This intrinsic lack of antigen presentation might be overcome by redirecting T cells to a tumor-associated antigen with a chimeric antigen receptor (CAR).

The inhibitory Notch ligand Delta-like protein 3 (DLL3) (6), a direct transcriptional target of the neuroendocrine transcription factor ASCL1 (7), is expressed specifically on the surface of neuroendocrine cancers, including SCLC (8). Low-level DLL3 mRNA expression has been detected in pituitary, brain, and testis (9) but, importantly, not on the cell surface of nonmalignant cells (8), making it an attractive tumor-specific target for antibody-drug conjugates (8, 10), bispecific T cell engagers (11, 12), and CAR T cells.

CAR T cell therapy has been highly successful against certain hematological malignancies; however, it has been much less so in patients with solid cancers to date. Various factors may contribute to a lack of CAR T cell effectiveness, including heterogeneous expression of the target antigen and an immunosuppressive tumor microenvironment (TME) (13). CAR T cells that secrete other factors such as cytokines may be used to alter this microenvironment. We and others have studied the impact of several cytokines in the context of CAR T cells, including the proinflammatory cytokines IL-12 and IL-18, both normally produced by macrophages. CAR T cells that secrete IL-12 have demonstrated evidence of increased activity and persistence in various hematologic and solid tumor models, including increased resistance to Treg-mediated inhibition in vitro and enhanced cytotoxicity and antitumor efficacy in vivo (14–19). IL-18 production by CAR T cells can also lead to enhanced antitumor efficacy and altered TME in immunocompetent mouse models (20–22).

In this study, we present the selection of an effective CAR against DLL3-expressing tumors. The DLL3-targeting CAR T cells were effective in xenograft and syngeneic SCLC models, and their activity was greatly enhanced with the secretion of IL-18. IL-18 stimulated persistence and activation of both CAR T cells and tumor-reactive endogenous T cells, as well as myeloid cells in the TME. Finally, human IL-18–secreting CAR T cells targeting DLL3 displayed potent antitumor efficacy in several xenogeneic SCLC models, an effect that could be further enhanced by ICB.

## Results

### Selection of optimal SC16-based single-chain variable fragment.

**Figure 1:**
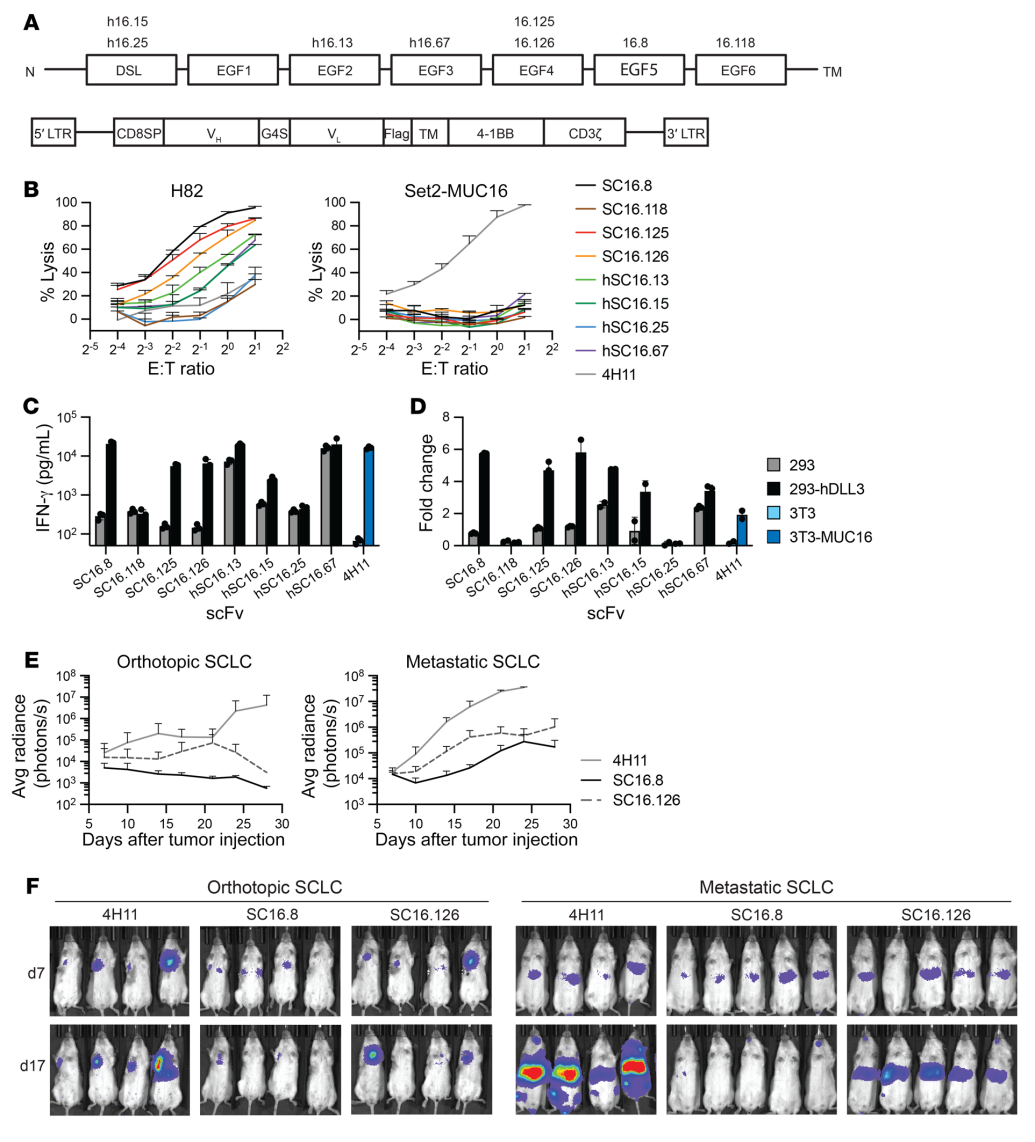
Selection of scFv for effective CAR T cells against DLL3 on small cell lung cancer. (**A**) Schematic of extracellular DLL3 domains with binding location of anti-DLL3 SC16 antibody clones (top), and schematic of CAR design for initial selection of single-chain variable fragments (bottom). LTR, long terminal repeats; CD8SP, CD8 signal peptide; V_H_, heavy chain; V_L_, light chain; TM, transmembrane domain; h, humanized. (**B**) Luciferase killing assay with CAR T cells cocultured with DLL3-expressing H82-SCLC or DLL3^–^ Set2 cells. The Set2 cells overexpress MUC16 as positive control for the MUC16-targeting 4H11 CAR (*n* = 3). (**C** and **D**) DLL3-specific activation of the SC16.8, SC16.125, and SC16.126 CARs in cocultures with DLL3^+^ 293 cells, (**C**) as measured by IFN-γ production (see also Supplemental Figure 1 for IL-2, GM-CSF, and TNF-α levels) and (**D**) 7-day proliferation (*n* = 3). 3T3-MUC16 cells were used as positive control for the 4H11 CAR. (**E** and **F**) Orthotopic or metastatic H82-SCLC tumor growth following administration of the indicated CAR T cells (*n* = 4–5).

Previous work has identified a series of high-affinity monoclonal antibodies (SC16) with binding specificity to distinct extracellular domains of DLL3 (Figure 1A). We initially sought to develop a highly functional CAR against DLL3 by cloning single-chain variable fragments (scFvs) derived from heavy- and light-chain pairs of these antibodies into a retroviral CAR vector with Flag-tag for easy detection (Figure 1A). This vector contained the 4-1BB costimulation domain. We tested scFvs derived from SC16 monoclonal antibody clones 8, 13, 15, 25, 67, 118, 125, and 126, and isolated T cells from healthy donors were transduced with each CAR. The lysis potential of CAR T cells was measured with a 20-hour luciferase killing assay with DLL3^+^ H82-SCLC cells. The 4H11 CAR, targeting the MUC16 protein (23), was used as negative control; Set2 cells (DLL3^–^ and MUC16^+^) also served as controls for specificity. None of the DLL3 CARs showed lysis of Set2 cells. There was substantial variation in H82 lysis capacity of the SC16 CARs, with SC16.8, SC16.125, and SC16.126 performing the best (Figure 1B).

We then used coculture experiments to assess DLL3-mediated CAR T cell activation based on cytokine release and proliferative response. For these experiments we utilized HEK293 cells (DLL3^–^) and HEK293 cells expressing human DLL3. The cytokines IFN-γ, IL-2, GM-CSF, and TNF-α were measured in the supernatant 24 hours after coculture, and CAR T cell proliferation was quantified after 7 days (Figure 1, C and D, and Supplemental Figure 1; supplemental material available online with this article; https://doi.org/10.1172/JCI166028DS1). We observed DLL3-independent activation of humanized SC16.13 (hSC16.13) and hSC16.67 CAR T cells and minimal activation of SC16.118 and hSC16.25 CAR T cells. Because the SC16.125 and SC16.126 CARs behaved similarly and both bind to the DLL3 EGF4 domain, we chose to proceed with SC16.8 and SC16.126 as lead candidates for in vivo testing.

1 × 10^6^ H82 cells were injected either in the lung parenchyma or tail vein of NSG mice. After 7 days mice were treated intravenously with 5 × 10^6^ CAR T cells. T cells expressing either the SC16.8 or SC16.126 CAR showed an antitumor response in both the orthotopic and metastatic SCLC model, compared with the negative control CAR 4H11 (Figure 1, E and F). SC16.8 CAR T cells performed better than SC16.126 in both contexts. Thus, of our panel of eight SC16 clones, SC16.8 CAR T cells demonstrated the best DLL3-specific activation and antitumor efficacy.

### IL-18 increases CAR T cell efficacy in a murine metastatic SCLC model.

**Figure 2:**
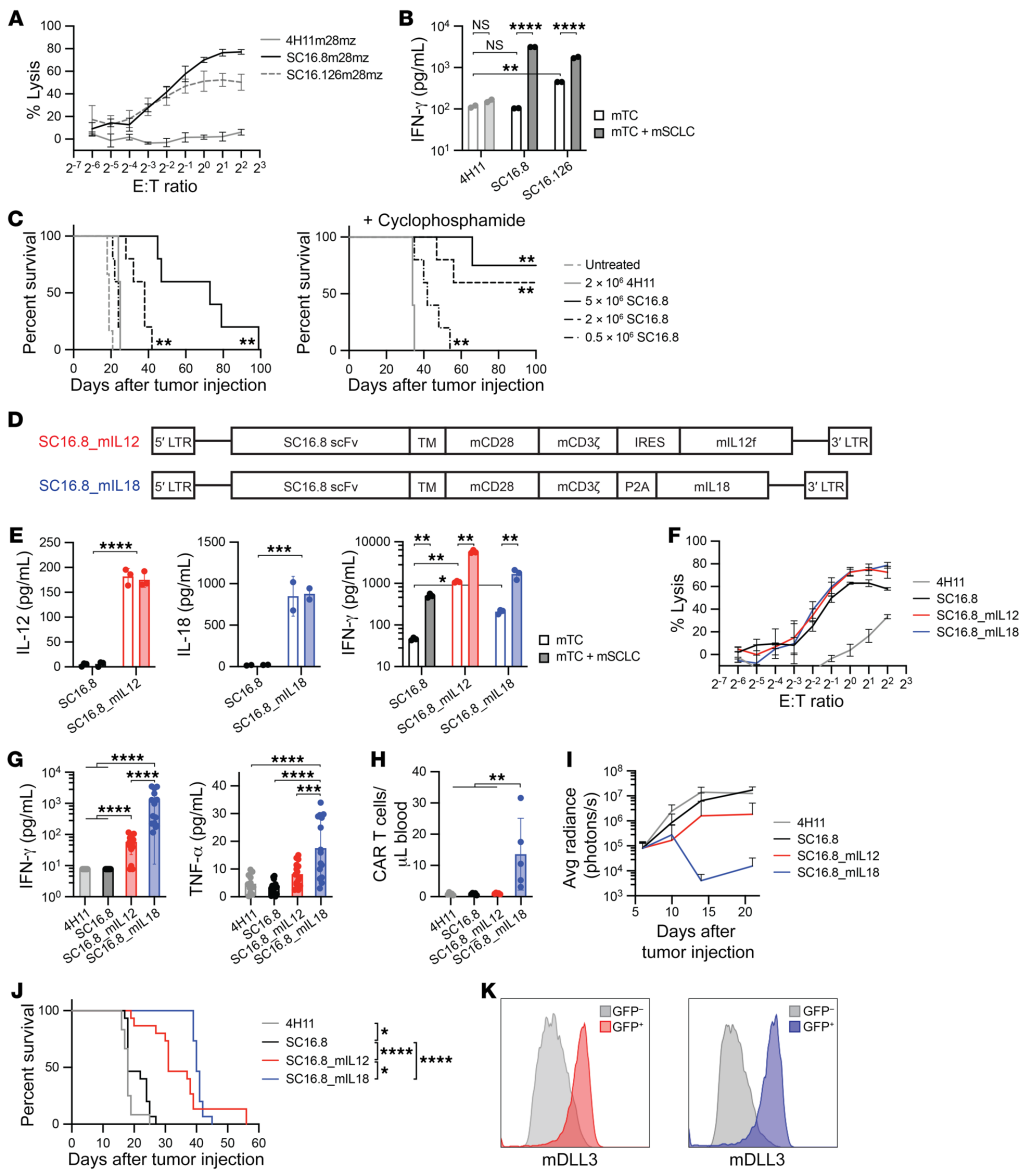
CAR T cell–secreted IL-12 and, especially, IL-18 prolong survival in murine SCLC model. (**A**) Luciferase killing assay with murine CAR T cells and mSCLC cells that endogenously express murine DLL3 (*n* = 3). The CAR consists of the SC16.8 single-chain variable fragment (scFv) and murine CD28 and CD3ζ domains. (**B**) IFN-γ levels in supernatant of 24-hour coculture of murine CAR T cells with or without mSCLC cells. **P < 0.01, ****P < 0.0001 (2-way ANOVA, *n* = 2). (**C**) Survival of mice with metastatic mSCLC that were treated with the indicated doses of SC16.8m28mz CAR T cells (day 7) with or without 50 mg/kg cyclophosphamide pretreatment (day 6). **P < 0.01 (log-rank test, *n* = 4–6). (**D**) Schematic of CAR design with SC16.8 scFv, murine CD28 and CD3ζ signaling domains, and the murine *Il12* fusion transgene or murine *Il18* transgene. IRES, internal ribosome entry site. (**E**) IFN-γ production by the respective CAR T cells in the absence and presence of mSCLC cells. *P < 0.05, **P < 0.01, ***P < 0.001, ****P < 0.0001 (2-way ANOVA and Student’s *t* test, *n* = 2–3). (**F**) Luciferase killing assay with SC16.8 CAR T cells that express IL-12, IL-18, or no cytokine and 4H11 CAR T cells as negative control (*n* = 3). (**G** and **H**) Serum IFN-γ and TNF-α levels (*n* = 12–15) and circulating CAR T cells (*n* = 5) in the blood of mice collected 3 days after treatment with the indicated CAR T cells. **P < 0.01, ***P < 0.001, ****P < 0.0001 (1-way ANOVA). (**I**) Tumor growth in mice that were treated with 2 × 10^6^ CAR T cells intravenously 7 days after systemic mSCLC administration (*n* = 12–15). (**J**) Survival of mice in **I**. *P < 0.05, ****P < 0.0001 (log-rank test). (**K**) DLL3 surface expression on end-stage tumors from mice treated with SC16.8m28mz_mIL12 (red) or SC16.8m28mz_mIL18 (blue) CAR T cells.

We next sought to explore the activity of these CAR T cells in an immunocompetent murine model, using a murine SCLC (mSCLC) cell line derived from a genetically engineered mouse model with concomitant loss of *Rb1* and *Trp53*. First, we tested whether the SC16.8 and SC16.126 CARs were cross-reactive to murine DLL3 by measuring the tumor lysis potential (Figure 2A) and IFN-γ production (Figure 2B) in coculture with mSCLC cells. Both CARs were activated by murine DLL3, but SC16.8 CAR T cells showed more lysis and higher target-specific IFN-γ levels. We proceeded with in vivo testing of SC16.8 CAR T cell functionality in a metastatic mSCLC model in immunocompetent mice based on tail vein injection of 1 × 10^6^ mSCLC allograft tumor cells. We titrated the amount of CAR T cells administered 7 days after systemic tumor cell injection with or without preconditioning chemotherapy of 50 mg/kg cyclophosphamide on day 6 (Figure 2C). High doses of CAR T cells alone led to improved survival. Preconditioning chemotherapy induced long-term survival in several mice receiving 2 × 10^6^ or 5 × 10^6^ CAR T cells and improved survival of mice receiving a low dose of 0.5 × 10^6^ CAR T cells, compared with negative control 4H11 CAR T cells.

To investigate the impact of IL-12 or IL-18 secretion by the CAR T cells on antitumor efficacy, we cloned murine *Il12* fusion or *Il18* transgenes into a retroviral vector with SC16.8m28mz CAR (Figure 2D). We validated the secretion of murine IL-12 (mIL-12) and murine IL-18 (mIL-18) in the supernatant of murine CAR T cells alone or cocultured with mSCLC cells (Figure 2E). Functionality of the 2 cytokines was confirmed by the increase in IFN-γ in unstimulated CAR T cells, which further increased in the presence of mSCLC cells (Figure 2E, right). As we have seen with other CARs, the lysis potential of all SC16.8 CAR T cell derivatives was similar, regardless of secreted cytokines (Figure 2F).

Next, we treated mice with systemic mSCLC tumors with 2 × 10^6^ CAR T cells in the absence of preconditioning chemotherapy. Three days later whole blood and sera were collected for flow cytometry and cytokine analysis. We observed a sharp increase in IFN-γ levels in the sera of mice treated with SC16.8 CAR T cells expressing mIL-12 (SC16.8_mIL12) or mIL-18 (SC16.8_mIL18), with the highest level when mIL-18 was secreted (Figure 2G). IFN-γ levels in 4H11 and SC16.8 CAR T cell–treated mice were below the level of detection. TNF-α levels were increased in the sera of mice that received SC16.8_mIL18 CAR T cells (Figure 2G). We were able to detect circulating CAR T cells only in the blood of mice treated with SC16.8_mIL18 CAR T cells (Figure 2H). In addition, only mIL-18–secreting CAR T cells were able to shrink mSCLC tumors (Figure 2I), which resulted in a prolonged survival of mice (Figure 2J). We did not observe antigen loss, as surface expression of mDLL3 was still detected on end-stage tumors that progressed after treatment with SC16.8_mIL12 or SC16.8_mIL18 CAR T cells (Figure 2K). In conclusion, mIL-18 secretion by CAR T cells improved the efficacy of DLL3-targeting CAR T cells in a murine metastatic SCLC model.

### mIL-18 stimulates CAR T cell proliferation and activates both CAR and endogenous T cells.

**Figure 3:**
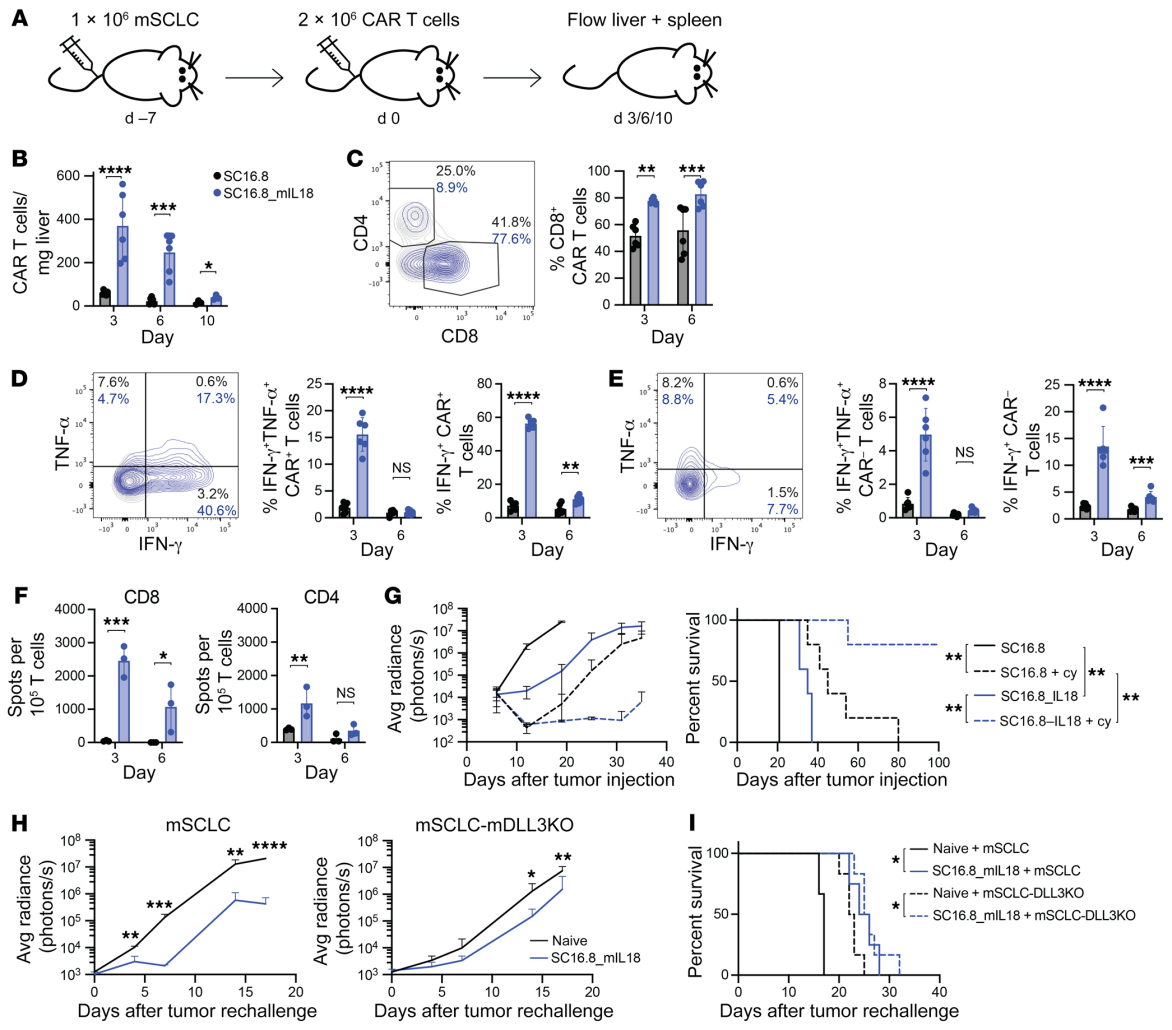
IL-18 increases activation of both genetically engineered and endogenous T cells. (**A**) Experimental setup for **B**–**E** and Figure 4, A–H. See also Supplemental Figure 2, A–C. 2 × 10^6^ murine mCherry-expressing SC16.8m28mz or SC16.8m28mz_mIL18 CAR T cells were administered 7 days after systemic mSCLC injection. 3, 6, or 10 days later livers and spleens were harvested for flow cytometry analysis (day 3 and day 6, *n* = 6 from 2 independent experiments; day 10, *n* = 3). (**B**) Levels of CAR T cells detected in the liver over time. (**C**) Example (day 3) and quantification of the CD8^+^ CAR T cell population. (**D** and **E**) Example (day 3) and quantification of (**D**) intracellular IFN-γ and TNF-α in CAR^+^ T cells and (**E**) CAR^–^ endogenous T cells in the liver. (**F**) CD4^+^ or CD8^+^ mCherry^–^ endogenous T cells were sorted on day 3 and 6 after CAR T cell treatment and cocultured with mSCLC, and IFN-γ release was measured with an ELISpot assay (*n* = 3). (**B**–**F**) *P < 0.05, **P < 0.01, ***P < 0.001, ****P < 0.0001 (2-way ANOVA). (**G**) Systemic mSCLC growth curve (left) and survival curve (right). Tumor-bearing mice were treated with low-dose 50 mg/kg cyclophosphamide (day 6) followed by low-dose 0.5 × 10^6^ CAR T cells (day 7). **P < 0.01 (log-rank test, *n* = 5). (**H**) Tumor growth of wild-type (*n* = 3–4) or DLL3KO mSCLC (*n* = 6, see Supplemental Figure 2D) in long-term surviving mice after cyclophosphamide plus SC16.8m28mz_mIL18 treatment with no evidence of tumor on day 42 (see **G**) compared with age-matched naive control mice. *P < 0.05, **P < 0.01, ***P < 0.001, ****P < 0.0001 (Student’s *t* test). (**I**) Survival of mice in **H**. *P < 0.05 (log-rank test, *n* = 6).

To study the effect of mIL-18 on CAR T cells and the TME we collected livers (main tumor location) and spleens for flow cytometry analysis at various time points after CAR T cell treatment (Figure 3A). We cloned mCherry into the retroviral vector for detection of transduced CAR T cells, to prevent potential undercounting because of CAR downregulation, and validated the antitumor efficacy of the mCherry^+^ CAR T cells in vivo (Supplemental Figure 2, A–C). mIL-18 induced more than 4-fold increase in the peak amount of CAR T cells in the liver, with a gradual reduction over time (Figure 3B). Because 10 days after treatment relatively few CAR T cells were detected, we focused on days 3 and 6 for further analysis of the T cells. SC16.8_mIL18 CAR T cells in the liver were enriched for CD8^+^ (Figure 3C). In addition to the increased number of SC16.8_mIL18 CAR T cells, we also observed increased activation, as quantified by intracellular staining for the effector cytokines IFN-γ and TNF-α (Figure 3D). We measured more IFN-γ^+^TNF-α^+^ double-positive cells on day 3 when the CAR T cells secrete mIL-18 and an increased percentage of total IFN-γ^+^ CAR T cells on days 3 and 6. Intriguingly, a similar pattern of increased in IFN-γ^+^TNF-α^+^ and total IFN-γ^+^ cells was present in the CAR^–^ bystander T cells (Figure 3E). We then assessed whether the activated bystander T cells would demonstrate tumor reactivity. Endogenous mCherry^–^ T cells were sorted from tumor-bearing livers 3 and 6 days after treatment with mCherry^+^ SC16.8 or SC16.8_mIL18 CAR T cells. Sorted CD4^+^ and CD8^+^ T cell populations were cocultured with mSCLC cells in an IFN-γ ELISpot assay (Figure 3F). CAR T cell–secretion of mIL-18 increased recruitment of tumor-specific CD4^+^ (on day 3) and especially CD8^+^ (on days 3 and 6) endogenous T cells. Sorted SC16.8_mIL18 CAR T cells retained their ex vivo antitumor cell response to a greater degree and longer than SC16.8 CAR T cells (Supplemental Figure 2, D and E).

Because 2 × 10^6^ SC16.8_mIL18 CAR T cells did not lead to long-term survival in this aggressive mSCLC model when administered 7 days after tumor injection (Figure 2J), we tested whether cyclophosphamide pretreatment would sensitize the tumors to mIL-18–secreting CAR T cells. Indeed, we observed a dramatic increase in antitumor response when the mSCLC-bearing mice received cyclophosphamide 1 day prior to a low dose of 0.5 × 10^6^ CAR T cells. SC16.8_mIL18 CAR T cells were now able to induce long-term survival (Figure 3G). To assess whether SC16.8_mIL18 CAR T cells are still present and active against tumor cells, we rechallenged mice on day 42 that did not have detectable residual tumor after combination treatment of cyclophosphamide plus 0.5 × 10^6^ SC16.8_mIL18 CAR T cells. These mice were not fully protected against mSCLC outgrowth, but the tumor growth was delayed compared with tumor growth in naive age-matched control mice (Figure 3H, left), suggestive of residual presence and activity of tumor-reactive T cells. We also assessed whether the increased activation of tumor-reactive bystander T cells (Figure 3, E and F) would lead to antitumor efficacy of these T cells independent of the CAR T cell response against mDLL3. Surviving mice with no evidence of tumor were rechallenged with mSCLC cells in which mDLL3 was knocked out (Supplemental Figure 2F). Again, the tumor outgrowth was slightly delayed (Figure 3H, right), consistent with an endogenous T cell response against other mSCLC antigens. Survival of the rechallenged mice with either wild-type mSCLC or mSCLC-mDLL3KO was prolonged compared with the survival of naive mice (Figure 3I). All together, these results demonstrate that the secretion of mIL-18 enhances the antitumor response of both CAR and endogenous T cells.

### mIL-18 reprograms and activates the myeloid compartment of the TME.

**Figure 4:**
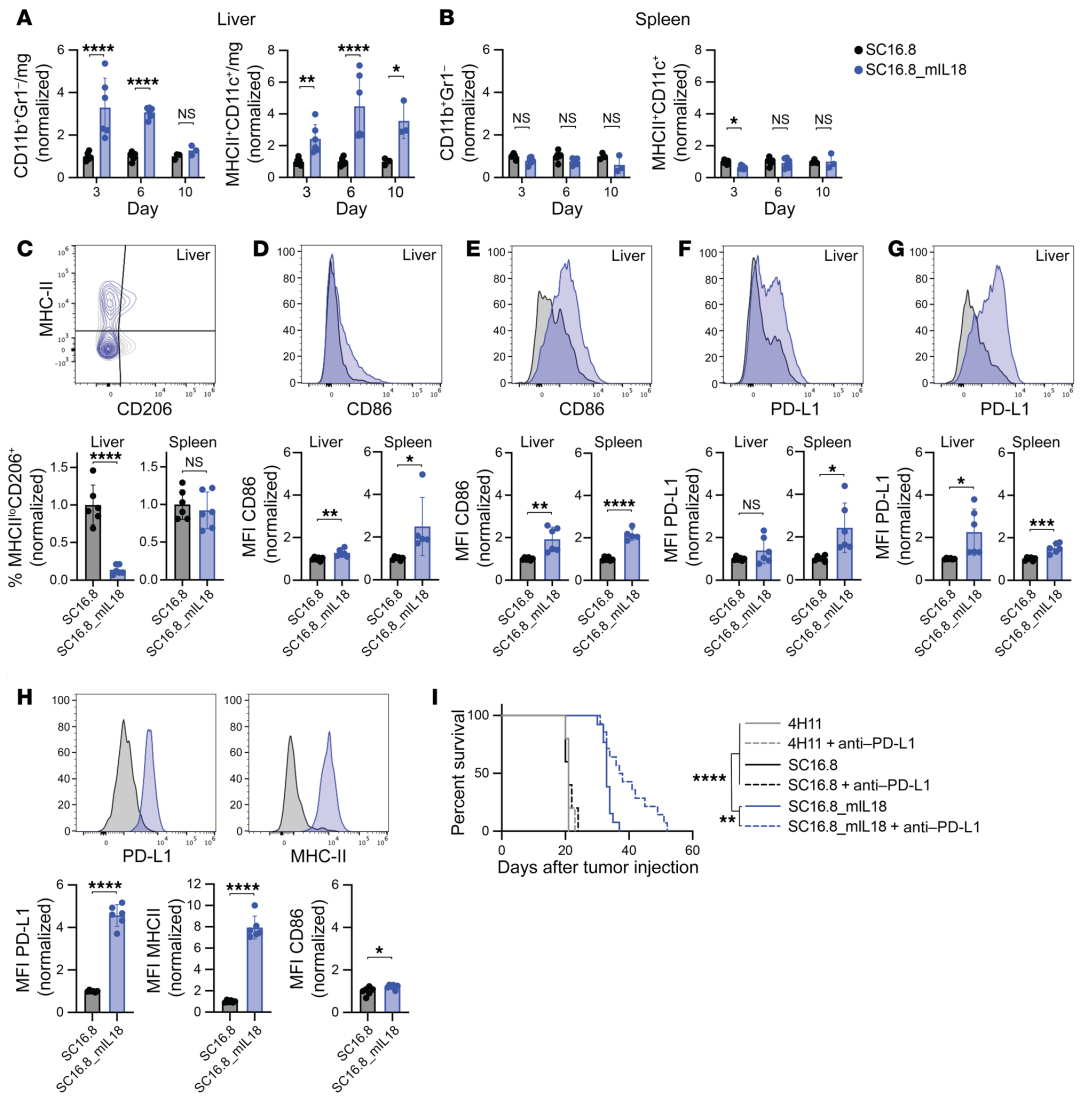
IL-18 recruits and reprograms myeloid cells in the tumor microenvironment. (**A** and **B**) Levels of CD11b^+^Gr1^–^ macrophages and MHC-II^+^CD11c^+^ dendritic cells detected (**A**) in the liver TME and (**B**) in the spleen 3, 6, and 10 days after CAR T cell treatment. *P < 0.05, **P < 0.01, ****P < 0.0001 (2-way ANOVA). (**C**–**H**) Representative flow plots and quantification on day 6 after CAR T cell administration. See Supplemental Figure 3 for quantification on day 3, 6, and 10. *P < 0.05, **P < 0.01, ***P < 0.001, ****P < 0.0001 (Student’s *t* test). (**C**) Levels of CD206^+^MHC-II^lo^ M2-like macrophages. (**D** and **E**) Expression of activation marker CD86 on (**D**) macrophages and (**E**) dendritic cells in liver TME and spleen. (**F** and **G**) Expression of checkpoint molecule PD-L1 on (**F**) macrophages and (**G**) dendritic cells in liver TME and spleen. (**H**) Expression of PD-L1, MHC-II, and CD86 on F4/80^+^ macrophages in the liver TME. (**I**) Survival of mice with metastatic mSCLC that received 2 × 10^6^ CAR T cells on day 7 and 250 μg anti–PD-L1 antibody on days 14, 17, and 20. **P < 0.01, ****P < 0.0001 (log-rank test, *n* = 5 for 4H11 and SC16.8, *n* = 14 for SC16.8_mIL18).

We analyzed the TME and spleen for levels and activation of macrophages and dendritic cells on the same days as described in Figure 3A. We observed an increase in both CD11b^+^Gr1^–^ macrophages and CD11c^+^MHC-II^+^ dendritic cells of live CD45^+^ cells in tumor-bearing livers of mice treated with mIL-18–secreting CAR T cells, compared with CAR T cells without mIL-18 (Figure 4A). No significant changes in prevalence of either cell type were seen in the corresponding spleens of these animals (Figure 4B). Importantly, liver macrophages of mice treated with mIL-18–secreting CAR T cells displayed a reduction in the CD206^+^MHC-II^lo^ “M2-like” antiinflammatory phenotype (Figure 4C and Supplemental Figure 3A). Macrophages and dendritic cells in both the liver and spleen expressed higher levels of activation marker CD86 (Figure 4, D and E, and Supplemental Figure 3, B and C), indicating a systemic activation of antigen-presenting cells. IFN-γ is known to induce PD-L1 expression. In line with our previous observation that mIL-18 secretion leads to higher IFN-γ levels in the serum (Figure 2G), we found an increase in PD-L1 levels on macrophages and dendritic cells following treatment with mIL-18–secreting CAR T cells (Figure 4, F and G, and Supplemental Figure 3, D and E). This increase in PD-L1 was especially profound on a subset of macrophages that express F4/80 (Figure 4H and Supplemental Figure 3F). The F4/80^+^ macrophages did not increase in number but were highly activated with increased expression of MHC-II and CD86 (Figure 4H and Supplemental Figure 3F).

PD-L1 expression in the TME, on tumor cells, or both, can hamper an effective CAR T cell antitumor response. We therefore assessed the potential benefit of blocking the inhibitory PD-L1/PD-1 interaction by combining CAR T cell treatment with anti–PD-L1 antibody therapy. Tumor-bearing mice received 3 doses of 300 μg anti–PD-L1 antibody starting 1 week after CAR T cell treatment. We observed a significant survival improvement when mIL-18–secreting CAR T cells were combined with anti–PD-L1 antibodies, whereas anti–PD-L1 therapy had no effect on survival following administration of CAR T cells that did not secrete mIL-18 (Figure 4I).

In conclusion, we have shown that the secretion of mIL-18 by DLL3-targeted CAR T cells leads to an activated, more proinflammatory myeloid compartment, both in the TME and systemically. The antitumor response can be further improved by blocking inhibitory effects of mIL-18–induced PD-L1 with antibodies.

### IL-18–secreting human DLL3-targeting CAR T cells cure mice with metastatic SCLC.

**Figure 5:**
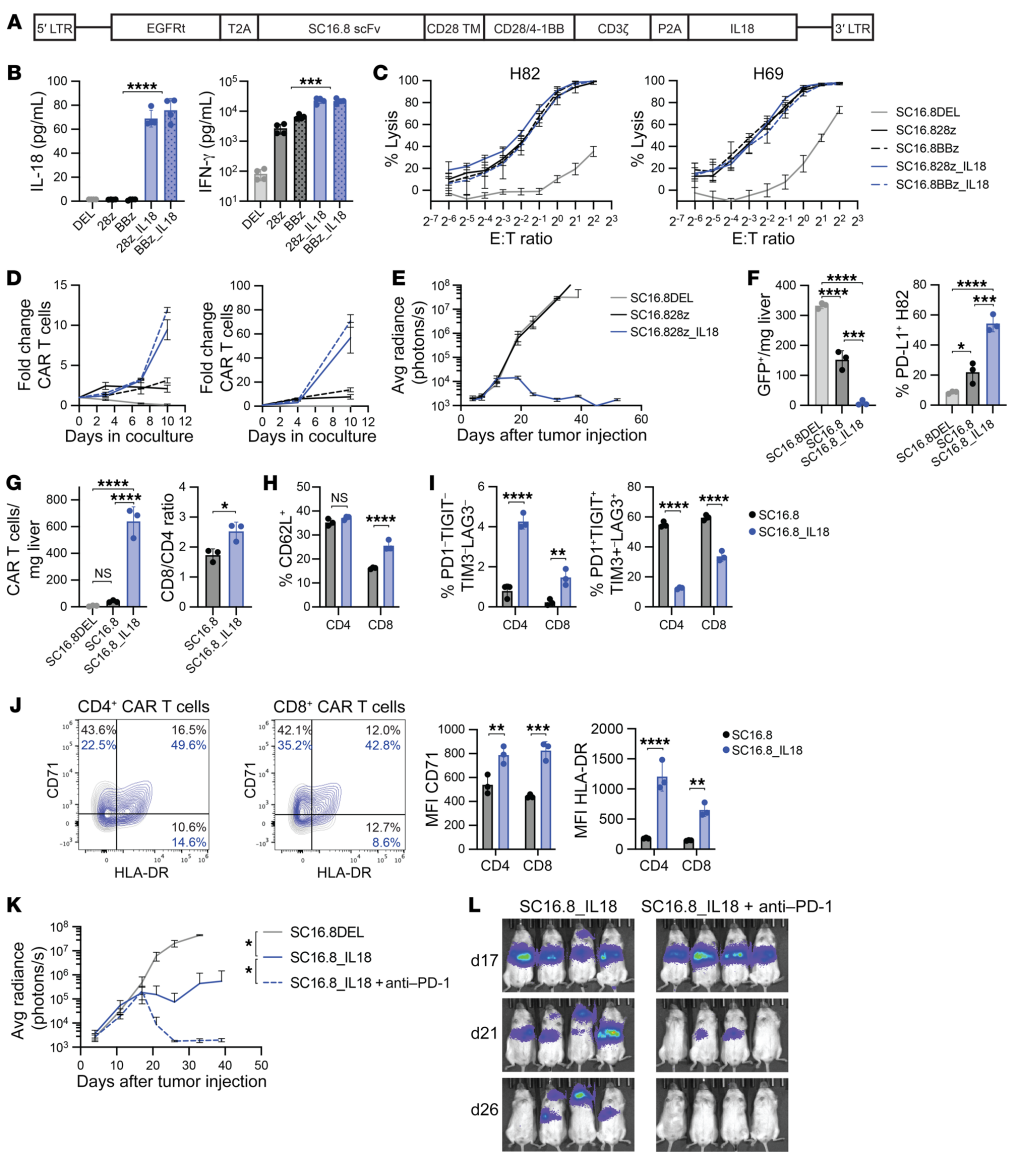
Human DLL3-targeting CAR T cells that secrete IL-18 are effective against SCLC and can be further potentiated by late-onset anti–PD-1 therapy. (**A**) Schematic overview of the CAR design. EGFRt, truncated EGFR; scFv, single-chain variant fragment. (**B**) Levels of IL-18 and IFN-γ in cell culture supernatant of cocultures of the indicated CAR T cells and DLL3-expressing target cells. ***P < 0.001, ****P < 0.0001 (Student’s *t* test, *n* = 4). (**C**) Luciferase killing assay with H82 (DLL3^+^) and H69 (DLL3^lo^) SCLC cells. (**D**) CAR T cell proliferation in cocultures with H82 or H69 SCLC cells (E/T ratio of 1:5, *n* = 3, representative result from 3 independent experiments). (**E**) Growth curve of metastatic H82-SCLC tumors in mice that received 1 × 10^6^ SC16.8 CAR T cells that did or did not secrete IL-18 (*n* = 4–5). (**F**–**J**) Livers of mice were subjected to flow cytometry analysis 9 days after CAR T cell treatment of mice with metastatic H82-SCLC tumors. *P < 0.05, **P < 0.01, ***P < 0.001, ****P < 0.0001 (2-way ANOVA, *n* = 3). (**F**) Cell count of GFP^+^ H82 tumor cells (left) and PD-L1 expression on these GFP^+^ cells (right). (**G**) Cell count of EGFRt^+^ CAR T cells in the liver (left) and CD8/CD4 ratio of the CAR T cells (right). (**H**) Quantification of CD62L^+^ central memory CAR T cells. (**I**) Quantification of CD4^+^ and CD8^+^ CAR T cells that are quadruple negative (left) or quadruple positive (right) for the exhaustion markers PD-1, TIGIT, TIM3, and LAG3. (**J**) Representative flow plots and mean fluorescence intensity (MFI) of T cell activation markers CD71 and HLA-DR on CAR T cells. (**K**) Growth curve of metastatic H82-SCLC tumors in mice that received 0.3 × 10^6^ CAR T cells alone or in combination with twice weekly 250 μg anti-human anti–PD-1 antibody, starting on day 18 (*n* = 4). (**L**) Bioluminescence imaging of same mice as in **K**, on days 17, 21, and 26 after tumor cell injection.

Having observed a marked improvement in the efficacy of our murine DLL3-targeting CAR T cells against systemic mSCLC in immunocompetent mice with CAR T cells secreting mIL-18, we sought to test the potential benefit of IL-18 on human CAR T cells in xenograft SCLC models. First, we cloned the human *IL18* transgene into a retroviral vector containing either the CD28 or the 4-1BB costimulatory domain and with a truncated EGFR for detection of transduced T cells (Figure 5A). The SC16.8DEL construct, which lacks the intracellular CD28/4-1BB and CD3ζ domains, was used as negative control. In the supernatant of cocultures with CAR T cells and DLL3-expressing cells, we validated the IL-18 expression as well as increased IFN-γ levels (Figure 5B). Lysis potential of the CAR T cells was similar, regardless of IL-18 secretion or costimulation with both H82 (DLL3^+^) and H69 (DLL3^lo^) SCLC cells (Figure 5C). In clinical trials, CAR T cell proliferation and expansion has proven an important hallmark of successful CAR T cell therapy. When we cocultured the SC16.8 CAR T cells with H82 or H69 SCLC cells (at an effector-to-target [E/T] ratio of 1:5), secreted IL-18 greatly enhanced the CAR T cell expansion in vitro (Figure 5D). Given the similarity in evident activity with either the CD28 or 4-1BB coactivation domains, we elected to further explore activity of the CD28 construct. Consistent with the augmentation of CAR T cell activity provided by endogenous IL-18 production, in vivo administration of 1 × 10^6^ SC16.8 CAR T cells failed to elicit an antitumor response, whereas the SC16.8_IL18 CAR T cells were able to fully eradicate H82 tumors (Figure 5E). Low doses of SC16.8_IL18 CAR T cells were able to prolong survival or induce long-term responses in SCLC models with metastatic H82 and H69 tumors and orthotopic SHP-77 tumors (Supplemental Figure 4).

We sought to further characterize the effects of IL-18 on human CAR T cell activation and phenotype in vivo. H82 tumor cells were infused 9 days prior to administration of 2 × 10^6^ CAR T cells, and livers (the main tumor location) were collected for flow cytometric analysis 9 days after treatment. The tumor burden was reduced in SC16.8 CAR T cell–treated mice, and it was even further reduced when treated with SC16.8_IL18 CAR T cells. Higher levels of PD-L1 were detected on the remaining tumor cells (Figure 5F). The number of CAR T cells detected in the liver was dramatically increased in the presence of IL-18. SC16.8DEL CAR T cell levels were so low that they could not be analyzed further. IL-18 secretion led to an increase in the CD8^+^/CD4^+^ CAR T cell ratio (Figure 5G). CD8^+^ CAR T cells displayed higher levels of the memory T cell marker CD62L (Figure 5H). We used a combination of 4 exhaustion markers (PD-1, TIGIT, LAG3, TIM3) to distinguish nonexhausted (quadruple negative) and fully exhausted (quadruple positive) CAR T cells. The secretion of IL-18 resulted in higher levels of nonexhausted and lower levels of fully exhausted CAR T cells (Figure 5I). The T cell activation markers CD71 and HLA-DR were more highly expressed on SC16.8_IL18 CAR T cells than on SC16.8 CAR T cells (Figure 5J). Finally, we wanted to test whether we could further increase the antitumor efficacy of SC16.8_IL18 CAR T cells with additional ICB. Mice with metastatic H82 tumors received a suboptimal dose of 0.3 × 10^6^ SC16.8_IL18 CAR T cells and were treated with 250 μg anti–PD-1 antibody twice weekly, starting 2 weeks after CAR T cell infusion, further improving the antitumor response of SC16.8_IL18 CAR T cells (Figure 5, K and L). These data, in a mouse lacking endogenous T cells, suggest a capacity for PD-1 blockade to further augment the efficacy of the anti-DLL3, IL-18–secreting CAR T cells directly.

All together, these results demonstrate that the secretion of IL-18 by SC16.8 CAR T cells increases a memory phenotype and T cell activation, reduces exhaustion, and improves the antitumor efficacy, which can be further enhanced by ICB in vivo.

## Discussion

CAR T cell therapies tested in patients with solid cancers have not proven to be highly effective, in contrast to hematological malignancies, for which 6 CAR T cell products are FDA approved (4 targeting CD19 and 2 targeting BCMA). Possible explanations for CAR T cell failure include an immunosuppressive TME leading to T cell exhaustion and heterogeneity of target antigen expression. Our IL-18–secreting CAR T cells against DLL3 might address both issues. In this study, we designed a CAR based on an anti-DLL3 SC16 antibody that is effective in various metastatic and orthotopic SCLC models with medium (H82 and SHP-77) and low (H69) surface expression levels of DLL3 (9). Other reports have shown that the proinflammatory cytokines IL-12 and IL-18, both normally produced by myeloid cells, stimulate CAR T cell activation and can alter the TME to a less immunosuppressive state. Different cytokines may be optimal in the context of different tumor types. Our CAR was cross-reactive to murine DLL3, which enabled us to study the effect of CAR T cell–secreted IL-12 and IL-18 in a syngeneic immunocompetent mSCLC model. In this direct comparison, IL-18 stimulated a more robust antitumor response than IL-12, which prompted us to further characterize the IL-18–induced changes to the TME.

In addition to enhanced activation of the CAR T cells themselves, endogenous tumor-infiltrating lymphocytes (TILs) were more activated in the presence of IL-18. Together with the increased antitumor TIL response in the ELISpot assay and delayed tumor outgrowth after rechallenge with DLL3KO mSCLC tumors, this suggests that IL-18 stimulates tumor-reactive TILs, which is in line with earlier findings in a CD19^+^ lymphoma model (20) and may be important for elimination of DLL3^–^ tumor cells in tumors with heterogeneous DLL3 expression. IL-18 secretion by the CAR T cells can shift an immunosuppressive microenvironment into a proinflammatory state (20, 22) by increasing the infiltration and activation of CD11b^+^Gr1^–^ macrophages and MHC-II^+^CD11c^+^ dendritic cells, both at the tumor site and systemically, and reducing CD206^+^MHC-II^lo^ M2-like polarization tumor-associated macrophages. SCLC tumors contain many macrophages (24), underlining the importance of repolarizing macrophages from an immunosuppressive to a proinflammatory state.

In addition to its effects on the TME, IL-18 strongly stimulated the proliferation, persistence, and memory phenotype of human DLL3 CAR T cells and reduced their exhaustion, leading to long-term remission in 3 different SCLC models. IL-18 is a potent inducer of IFN-γ production, which we observed in the sera of SC16.8_mIL18 CAR T cell–treated mice and in supernatant of human SC16.8_IL18 CAR T cells cocultured with DLL3-expressing target cells. IFN-γ, however, led to upregulation of the inhibitory ligand PD-L1 on both tumor cells and myeloid effector cells. Combination treatment of a suboptimal dose of IL-18–secreting CAR T cells with delayed ICB improved the CAR T cell response. ICB is approved for SCLC and integrated in first-line treatment. Potential benefits and timing of ICB after CAR T cell treatment will need to be tested in clinical trials. Similarly, the safety profile of IL-18–secreting CAR T cells will need to be studied. Importantly, no increase in IL-6, a cytokine associated with cytokine release syndrome, was observed in a murine CD19_mIL18 model (20), and no safety concerns were reported in the first patients that received huCART19-IL18 (25).

Given the typically poor clinical outcome, SCLC is in dire need of more targeted treatment options and CAR T cells may be a valuable addition. After preconditioning chemotherapy, which is routinely given prior to CAR T cell infusion, our SC16.8_mIL18 CAR T cells induced long-term remission in an aggressive immunocompetent metastatic mSCLC model. A recent study describes CAR T cells targeting AC133 on chemotherapy-resistant SCLC stem cells in combination with ICB and CD73 inhibition (26). This triple combination was effective in a xenograft SCLC model, but its activity has not yet been reported in a representative syngeneic mouse model.

DLL3 is expressed on a wide variety of neuroendocrine cancers, including high-grade neuroendocrine cancers of the lung, prostate, breast, pancreas, and intestinal tract as well as low-grade glioma and neuroblastoma. Neuroendocrine cancers often lack therapeutic options and most have a poor prognosis. Our potent DLL3-targeting CAR T cells could serve as an important addition to the treatment armamentarium for patients with SCLC and other DLL3^+^ neuroendocrine cancers.

## Methods

### Cell lines.

H82 (ATCC, HTB-175), H69 (ATCC, HTB-119), and SHP-77 (ATCC, CRL-2195) SCLC cells and murine *Trp53^–/–^;Rb^–/–^* mSCLC cells (provided by AbbVie and Julien Sage, Stanford University, Stanford, California, USA) were stably transfected with a retroviral vector expressing GFP and luciferase and were maintained in RPMI1640 medium (Gibco) supplemented with 10% heat-inactivated FBS, L-glutamine, and penicillin/streptomycin. Galv9 293 (courtesy of Michel Sadelain, Memorial Sloan Kettering Cancer Center [MSKCC], New York, New York, USA) and Phoenix ecotropic (ATCC, CRL-3214) packaging cells were maintained in DMEM (Gibco) supplemented with 10% FBS, L-glutamine, and penicillin/streptomycin.

### Animal models and treatments.

Six- to eight-week-old male NSG (NOD.Cg-Prkdc^scid^IL2rg^tm1Wjl^/SzJ) mice were purchased from The Jackson Laboratory (no. 005557) and used for all xenograft experiments. For the metastatic SCLC models, 1 × 10^6^ H82 or H69 cells in 200 μL PBS were injected into the tail vein. For the orthotopic SCLC models, 1 × 10^6^ H82 or SHP-77 cells in 40 μL PBS were injected intrathoracically. Tumor size was measured with bioluminescence for randomization before CAR T cell transfer and then once or twice per week to monitor tumor growth. Anti–PD-1 antibody (BioLegend, clone EH12.2H7, 250 μg i.p.) was given twice weekly for 3 weeks, starting 2 weeks after CAR T cell administration.

Six- to eight-week-old female immunocompetent B6129SF1/J mice (The Jackson Laboratory, no. 101043) were used for all experiments with mSCLC cells. 1 × 10^6^ mSCLC cells were injected in 200 μL PBS into the tail vein, and 7 days later mice received 2 × 10^6^ (unless indicated otherwise) murine CAR T cells. When indicated, mice received 1 i.p. injection of 50 mg/kg cyclophosphamide 1 day prior to CAR T cell transfer. Tumor size was measured with bioluminescence for randomization before CAR T cell treatment and then once or twice per week to monitor tumor growth. Anti–PD-L1 antibody (BioXCell, clone 10F.9G2, 300 μg i.p.) was given on days 14, 17, and 20 after tumor injection.

For the rechallenge with wild-type or DLL3KO mSCLC cells, B6129SF1/J mice received 1 × 10^6^ mSCLC cells via tail vein (day 0), 50 mg/kg cyclophosphamide i.p. (day 6), and 0.5 × 10^6^ SC16.8_mIL18 CAR T cells via tail vein (day 7). On day 42, mice without detectable tumor (~75% of cohort) and age-matched naive control mice received 1 × 10^6^ mSCLC or mSCLC-DLL3KO cells via tail vein.

### Generation of retroviral constructs.

Plasmids encoding the human and mouse CAR constructs in a SFG γ-retroviral vector (27) were used to transfect gpg29 fibroblasts (H29) with the ProFection Mammalian Transfection System (Promega) according to manufacturer’s instructions. The retroviral supernatants were used for transduction of Galv9 293 or Phoenix ECO cell lines to generate stable retroviral particle-producing cell lines. All vectors were generated by restriction enzyme digest and Gibson Assembly (New England BioLabs). For the human CARs, we used human EGFR cDNA without the intracellular domain, a T2A self-cleaving peptide, cDNA of the scFvs, the Flag-tag sequence (DYKDDDDK), the human CD28 transmembrane and intracellular domain, the human 4-1BB intracellular domain, the human CD3ζ intracellular domain, a P2A self-cleaving peptide, and the mature, processed form of human IL-18. For the murine CARs, we used mCherry, a T2A self-cleaving peptide, cDNA of the SC16.8 scFv with Flag-tag, the murine CD28 transmembrane and intracellular domain, the murine CD3ζ intracellular domain, an internal ribosome entry site, a fusion gene encoding the complete murine IL-12 with a serine-glycine repeat between the p35 and p40 chain-coding domains (provided by Alan Houghton and Jedd Wolchok, MSKCC), a P2A self-cleaving peptide, and the mature form of murine IL-18.

### T cell isolation and retroviral transduction.

Human and mouse T cells were isolated, activated, and transduced as described previously (28, 29). In brief, peripheral blood mononuclear cells were isolated from leukopacks (New York Blood Center, New York, New York, USA), from which T cells were isolated with a EasySep Human T Cell Isolation Kit (Stemcell Technologies). T cells were activated with CD3/CD28 Dynabeads (bead/cell ratio of 1:5) and 100 IU/mL IL-2 (Peprotech) and cultured in RPMI1640 supplemented with 10% FBS, 2 mM L-glutamine, and 1% penicillin/streptomycin. After 48 hours, CD3/CD28 beads were removed, and T cells were transduced by spinoculation on plates coated with retronectin (Takara Clontech) with retroviral supernatant from viral packaging cells. Mouse T cells were isolated from spleens, following red blood cell lysis, with a nylon wool fiber column (Polysciences). T cells were activated with CD3/CD28 Dynabeads (bead/cell ratio of 1:2) and 100 IU/mL IL-2 and cultured in RPMI1640 supplemented with 10% FBS, 2 mM L-glutamine, nonessential amino acids, sodium pyruvate, HEPES, 2-mercaptoethanol, and 1% penicillin/streptomycin. After 24 and 48 hours, T cells were transduced by spinoculation on retronectin-coated plates in viral supernatant from Phoenix ECO cells. T cells were rested for 1 day and then used for in vitro and in vivo assays.

### Cytotoxicity assays.

Mouse or human CAR T cells were cocultured at varying E/T ratios with 5 × 10^4^ firefly luciferase-expressing tumor cells in a 96-well plate in 200 μL media for 20 hours. 75 ng D-luciferin (Gold Biotechnologies) in 5 μL PBS was added to each well and luminescence was measured with a Tecan Sparke microplate reader (Tecan). Wells with target cells alone were used to determine the “max signal.” Percentage lysis was calculated as [1–(“sample signal”/“max signal”)] × 100.

### Proliferation assays.

1 × 10^5^ human CAR T cells were cocultured in a 12-well plate with 5 × 10^5^ target cells on day 0. At the indicated time points, each well was resuspended and 20 μL was taken for quantification of CAR T cells by flow cytometry using volume analysis with the Attune NxT flow cytometer (Thermo Fisher Scientific).

### Cytokine analysis.

For analysis of in vitro cytokine secretion, 1 × 10^5^ CAR T cells were cocultured with 1 × 10^5^ target cells in a 96-well plate in 200 μL media. After 24 hours, the supernatant was collected. For serum cytokine analysis, whole blood was collected and serum was prepared by allowing the blood to clot by centrifuging at 14,000*g* for 15 minutes at 4°C. Samples were analyzed with Milliplex Map Human cytokine/chemokine, Premixed 12-plex kit or the Milliplex Map Mouse cytokine/chemokine, Premixed 10-plex kit, for human and mouse cytokines respectively, and the FlexMap 3D system on a Luminex IS100 system (Millipore).

### Flow cytometry.

A Gallios B43618 (Beckman Coulter) and Attune NxT (Thermo Fisher Scientific) were used for flow cytometric data acquisition, and analysis was performed with FlowJo software. DAPI (0.5 mg/mL, Sigma-Aldrich) or a LIVE/DEAD fixable yellow dead cell stain kit (Thermo Fisher Scientific) was used to exclude dead cells in all experiments. CAR expression was detected with anti-Flag (L5), Cetuximab-A647, or mCherry. Tumor cells were detected with EGFP. DLL3, both human and mouse, was detected with a SC16.34-PE antibody (provided by AbbVie). Nonspecific binding of antibodies was blocked with anti-mouse CD16/CD32 antibody. The following anti-mouse antibodies were used: anti-CD45 (clone 30F11, BioLegend), anti-CD3ε (145-2C11, eBioscience), anti-CD4 (GK1.5, eBioscience), anti-CD8α (53-6.7, eBioscience), anti–TNF-α (MP6-XT22, BioLegend), anti–IFN-γ (XMG1.2, BioLegend), anti-CD11b (M1/70, eBioscience), anti-Gr1 (RB6-8C5, BioLegend), anti-CD11c (N418, eBioscience), anti–MHC-II I-A/I-E (M5/114.15.2, BioLegend), anti-CD206 (MMR, eBioscience), anti-F4/80 (BM8, eBioscience), anti-CD86 (GL1, BioLegend), and anti–PD-L1 (10F.9G2, BioLegend). For intracellular staining of TNF-α and IFN-γ, single-cell suspensions from livers and spleens were stimulated with 1× Cell Stimulation Cocktail (phorbol 12-myristate 13-acetate, ionomycin, brefeldin A, and monensin) from Thermo Fisher Scientific for 4 hours. Cells were then processed with the Cytofix/Cytoperm Plus kit (BD Biosciences). The following anti-human antibodies were used: anti-CD45 (30-F11, BioLegend), anti–PD-L1 (29E.2A3, BioLegend), anti-CD4 (SK3, eBioscience), anti-CD8 (SK1, BD Biosciences), anti-CD71 (CY1G4, BioLegend), anti–HLA-DR (L243, BioLegend), anti–PD-1 (EH12.2H7, BioLegend), anti-TIGIT (A15153G, BioLegend), anti-TIM3 (F38-2E2, BioLegend), anti-LAG3 (11C3C65, BioLegend), and anti-CD62L (DREG-56, BioLegend).

Cell isolation from livers and spleens and the preparation for flow cytometry were previously described (30). For analysis of circulating CAR T cells, whole blood was collected and spun at 14,000*g* for 15 minutes at 4°C. Red blood cell lysis was done with an ammonium-chloride-potassium Lysing Buffer (Lonza). Cells were washed with PBS and used for flow cytometric analysis.

### ELISpot assay.

A single-cell suspension of the liver of mice treated with 2 × 10^6^ CAR T cells was prepared and subjected to fluorescence-activated cell sorting (using a BD Aria cell sorter) as previously described (30). mSCLC cells were irradiated with 30 Gy using a GE Mark1 cesium irradiator, and 1 × 10^5^ cells were plated per well in a mIFN-γ ELISpot plate (Millipore ELISpot plates, Mabtech). Sorted T cell populations were added at a 1:1 effector-to-target ratio and incubated at 37°C overnight. After 24 hours, the plate was developed according to manufacturer’s instructions and analyzed with ImmunoSpot software.

### Generation of DLL3 knock out cells.

HEK-293FT cells were transfected using X-tremeGene HP (Sigma Aldrich) with a LentiCRISPRv2 vector encoding Cas9, sgRNA (CGGCTCGCACCGCGCGCGGT), and a puromycin resistance gene and the packaging plasmids psPAX2 and pMD2.G. HEK-293FT supernatants from 48 and 72 hours after transfection were pooled and used for transduction of mSCLC cells. After puromycin selection, single-cell clones were generated and upon outgrowth tested for DLL3 expression by flow cytometry.

### Statistics.

All statistical analyses were performed with GraphPad Prism software. Statistical significance was determined using an unpaired, 2-tailed Student’s *t* test, 1-way ANOVA, or 2-way ANOVA, as indicated in the figure legends. The log-rank (Mantel-Cox) test was used to determine statistical significance for overall survival in mouse survival experiments. Data are shown as the mean ± SD. *P* values of less than 0.05 were considered significant.

### Study approval.

Mice were housed under specific pathogen–free conditions in the animal facility of MSKCC, and all experiments were performed in accordance with MSKCC Institutional Animal Care and Use Committee–approved protocol guidelines (MSKCC 00-05-065, 13-07-007, and 18-06-009).

## Author contributions

JEJ and RJB conceptualized the study. JEJ and RJB provided methodology. JEJ, JFK, WDG, AVL and MC acquired data. JEJ analyzed data. CMR and RJB acquired funding. JEJ administrated the project. CMR and RJB supervised the study. JEJ wrote the original draft of the manuscript. CMR and RJB reviewed and edited the manuscript.

## Supplementary Material

Supplemental data

## Version 1 03/23/2023

In-Press Preview

## Version 2 05/01/2023

Electronic publication
