# Supplementary material for: IL-18–secreting CAR T cells targeting DLL3 are highly effective in small cell lung cancer models
Source: J Clin Invest. 2023 May 1;133(9):e166028. doi: 10.1172/JCI166028 (PMC10145930; doi:10.1172/JCI166028)
Supplement: Supplemental data [file jci-133-166028-s130.pdf]

Supplemental Figures

IL-18-secreting CAR T cells targeting DLL3 are highly effective in small cell lung cancer models

Janneke E. Jaspers[1], Jonathan F. Khan[2], William D. Godfrey[1], Andrea V. Lopez[1], Metamia Ciampricotti[1], Charles M. Rudin[1,3], Renier J. Brentjens[2,4*]

[1]Department of Medicine, Memorial Sloan Kettering Cancer Center; New York, NY, USA. [2]Weill Cornell School of Medicine; New York, NY, USA. [3]Thoracic Oncology, Memorial Sloan Kettering Cancer Center; New York, USA. [4]Department of Medicine, Roswell Park Comprehensive Cancer Center; Buffalo, NY, USA. *Corresponding Author: Renier J. Brentjens; Roswell Park Comprehensive Cancer Center; Elm and Carlton Streets; Buffalo, NY 14263; 716-845-2300; Renier.Brentjens@RoswellPark.org.

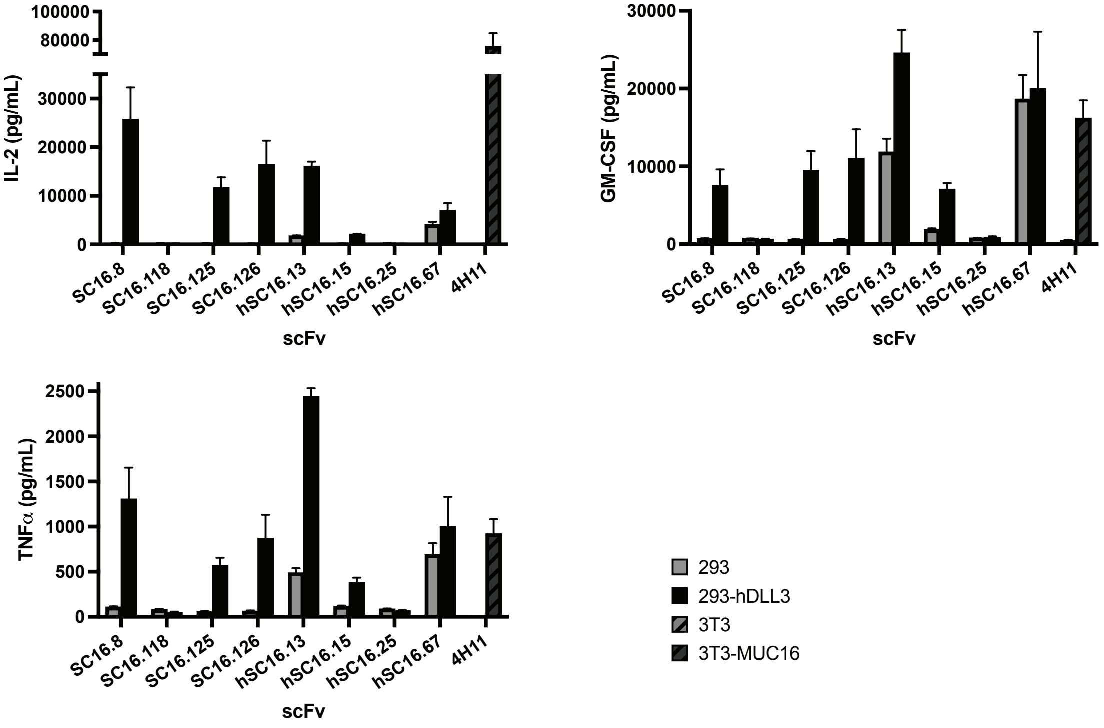

Supplemental Figure 1

Corresponding to Figure 1C. Cytokine levels of IL-2, GM-CSF and TNFα in co-cultures of CAR T cells with DLL3[+] 293 cells. 4H11 CAR T cells were co-cultured with 3T3 cells expressing the 4H11 target MUC16.

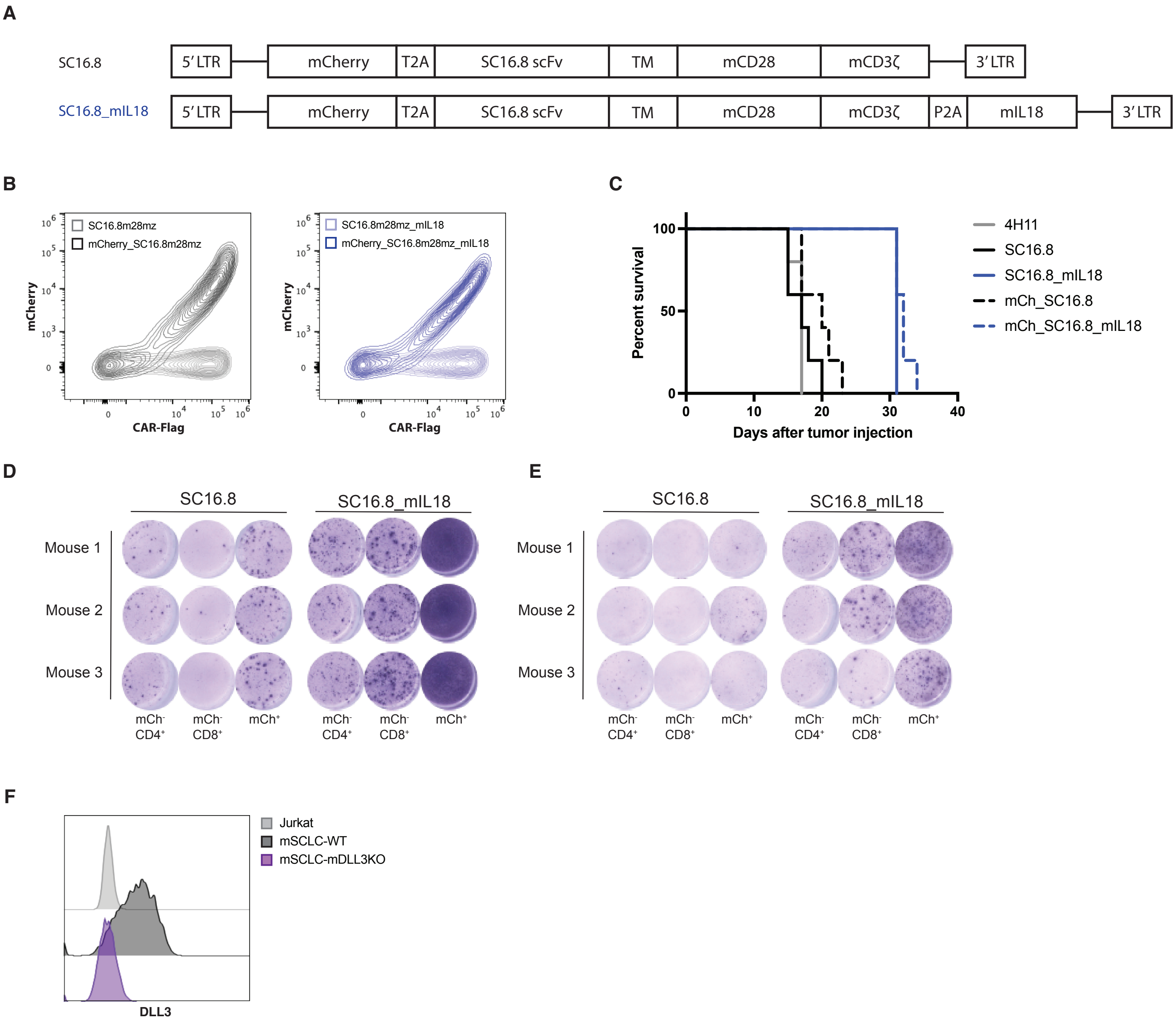

Supplemental Figure 2

Corresponding to Figure 3. **A)** Schematic of CAR design with mCherry, SC16.8 scFv, murine CD28 and CD3z signaling domains, and the murine IL-18 transgene. **B)** Flow cytometric analysis of murine CAR T cells for mCherry and CAR-Flag expression. **C)** Survival of mSCLC tumor-bearing mice after treatment with mCherry-expressing CAR T cells that do or do not secrete mIL-18, compared to the original mCherry-negative CAR T cells. **D)** and **E)** IFNg ELISPOT results, quantified in Figure 3F, comparing mCherry-negative endogenous CD4 and CD8 tumor-infiltrating lymphocytes from mice treated with SC16.8 or SC16.8_mIL18 CAR T cells, harvested from mSCLC-bearing livers 3 (**D**) or 6 (**E**) days after CAR T cell treatment. mCherry+ CAR T cells were isolated and included as positive control. **F)** DLL3 surface expression on DLL3-negative Jurkat cells, DLL3-positive mSCLC cells and DLL3-knockout mSCLC cells.

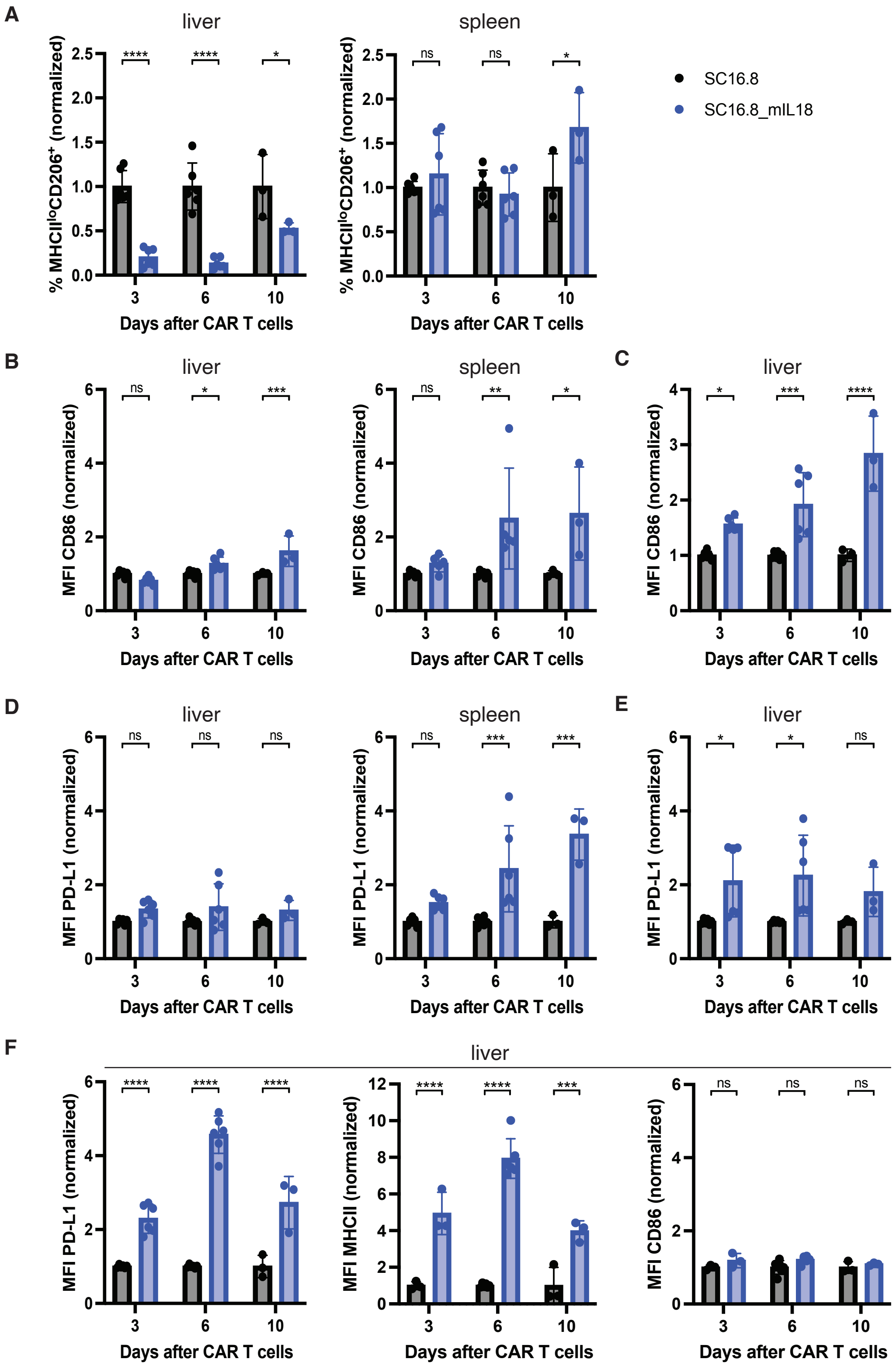

Supplemental Figure 3

Complete flow cytometric analysis of myeloid cells on days 3, 6 and 10 post CAR T cell treatment in mSCLC-bearing mice, corresponding to Figure 4. **A)** Levels of CD206^{+}MHC-II^{lo} M2-like macrophages in livers and spleens. **B)** Expression of CD86 on macrophages in livers and spleens. **C)** Expression of CD86 on dendritic cells in livers and spleens. **D)** Expression of PD-L1 on macrophages in livers and spleens. **E)** Expression of PD-L1 on dendritic cells in livers and spleens. **F)** Expression of PD-L1, MHC-II and CD86 on F4/80^{+} macrophages in livers.

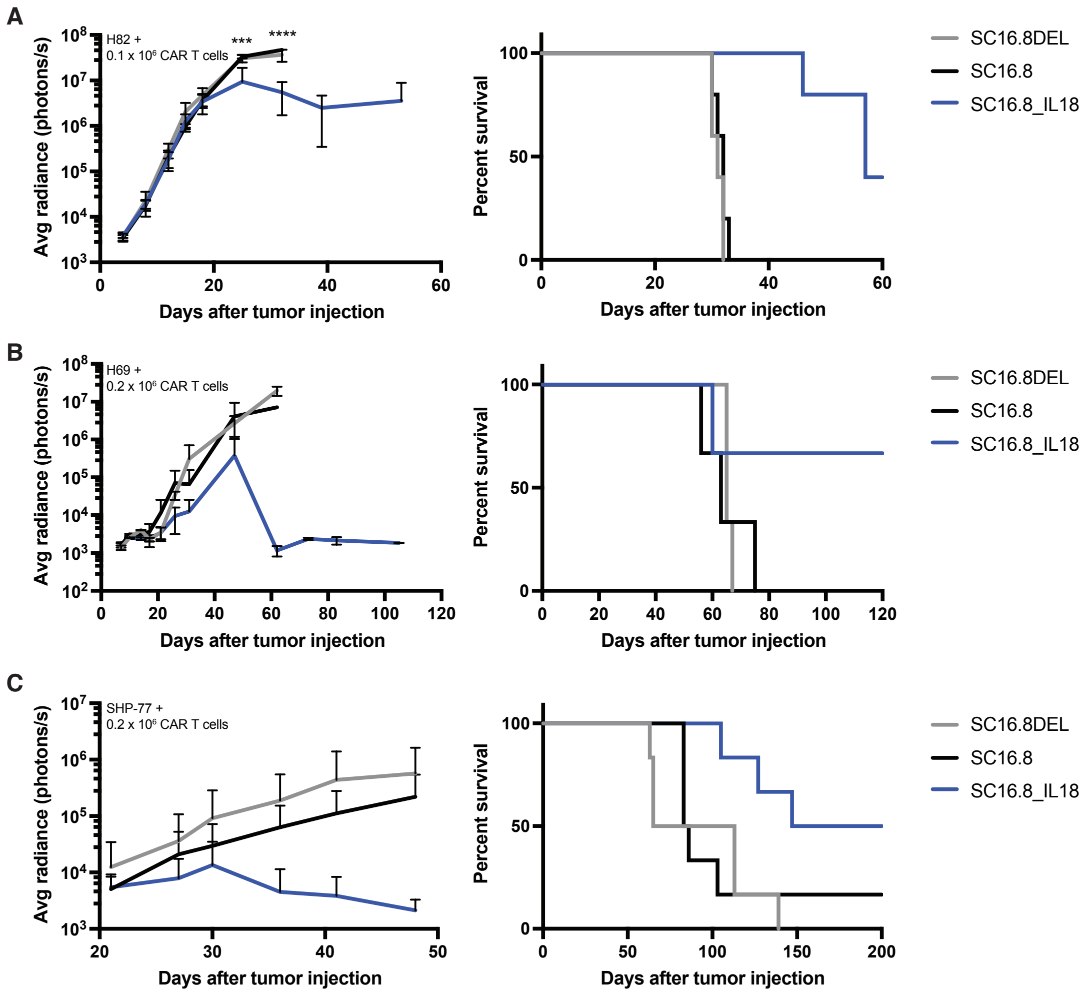

Supplemental Figure 4

Efficacy of low-dose SC16.8_IL18 CAR T cells in SCLC models, corresponding to Figure 5. **A)** Tumor growth and overall survival of mice with metastatic H82 tumors that received 0.1 x 10^6 CAR T cells. **B)** Tumor growth and overall survival of mice with metastatic H69 tumors that received 0.2 x 10^6 CAR T cells. **C)** Tumor growth and overall survival of mice with orthotopic SHP-77 tumors that received 0.2 x 10^6 CAR T cells.
